# Mesenchymal Stromal Cells Prevent Blood-induced Degeneration of Chondrocytes in a New Model of Murine Hemarthrosis

**DOI:** 10.1097/HS9.0000000000000924

**Published:** 2023-06-27

**Authors:** Alexandre Théron, Marie Maumus, Claire Bony-Garayt, Nicolas Sirvent, Christine Biron-Andreani, Christian Jorgensen, Danièle Noël

**Affiliations:** 1 IRMB, University of Montpellier, INSERM, Montpellier, France; 2 Resources and Competence Center for hereditary hemorrhagic diseases, CHU Montpellier, France; 3 Department of Pediatric Oncology and Hematology, CHU Montpellier, France; 4 Clinical Immunology and Osteoarticular Disease Therapeutic Unit, Department of Rheumatology, CHU Montpellier, France

## Abstract

Hemophilia is a rare congenital bleeding disorder caused by deficiency in coagulation factors VIII or IX, which is treated with prophylactic clotting factor concentrates. Nevertheless despite prophylaxis, spontaneous joint bleedings or hemarthroses still occur. The recurrent hemarthroses lead to progressive degradation of the joints and severe hemophilic arthropathy (HA) in patients with moderate and even mild forms of the disease. In absence of disease modifying treatment to stop or even delay HA progression, we aimed at evaluating the therapeutic potential of mesenchymal stromal cells (MSCs)-based therapy. We first developed a relevant and reproducible in vitro model of hemarthrosis relying on blood exposure of primary murine chondrocytes. We found that 30% whole blood for 4 days allowed to induce the characteristic features of hemarthrosis including low survival of chondrocytes, apoptosis induction, and dysregulation of chondrocyte markers in favor of a catabolic and inflammatory phenotype. We then evaluated the potential therapeutic effects of MSCs in this model using different conditions of coculture. Addition of MSCs improved the survival of chondrocytes when added either during the resolution or the acute phases of hemarthrosis and exerted a chondroprotective effect by enhancing the expression of anabolic markers, and reducing the expression of catabolic and inflammatory markers. We here provide the first proof-of-concept that MSCs may exert a therapeutic effect on chondrocytes under hemarthrosis conditions using a relevant in vitro model, thereby confirming a potential therapeutic interest for patients with recurrent joint bleedings.

## Graphical abstract

**Figure:**
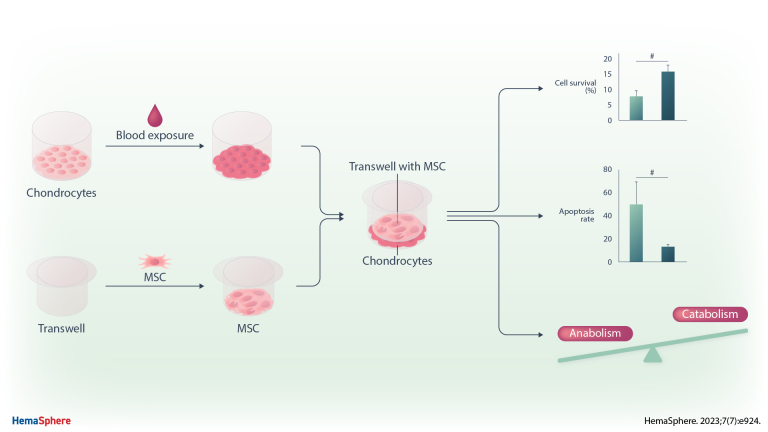

## INTRODUCTION

Hemophilia A and B are rare congenital bleeding disorders related to total or partial deficiency of coagulation factors VIII or IX, respectively. These are X-linked recessive disorders and the level of disease severity is based on the amount of residual activities of factor VIII or IX. In patients with severe or moderate hemophilia, and more rarely in patients with mild hemophilia, spontaneous bleeding occurs, primarily into large joints.^1^ Therefore, repeated articular bleedings or hemarthroses is a major complication of hemophilia leading to progressive degradation of the joints and severe hemophilic arthropathy (HA).^2^ Clinically and biologically, HA resembles osteoarthritis and rheumatoid arthritis with inflamed synovium, cartilage degeneration, and bone resorption directly provoked by the action of iron and blood in the joint. Although the introduction of prophylactic treatments has greatly reduced the occurrence of joint damage in the severe forms of hemophilia, a large proportion of patients still have affected joints, and patients on prophylaxis or with minor forms may also develop late arthropathy.^3,4^

Once the arthropathy is established, treatments are essentially symptomatic, based primarily on analgesics. They can be given by general route or local infiltration and act on the inflammatory part of the disease. In advanced stages, techniques such as synoviorthesis (radioisotopic or chemical synovectomy) and surgical synovectomy are used, but ultimately, total joint replacement is indicated.^5,6^ Other disease modifying treatments directed against pathophysiological targets including iron deposition, inflammation, hyperfibrinolysis, or bone remodeling are being evaluated in preclinical settings with promising results.^7^ However, translation into clinical practice is still far and there is room for other innovative therapeutic options.

Mesenchymal stromal cells (MSCs) are adult multipotent cells that have the capacity to differentiate into mature cells of the musculoskeletal system and secrete many factors, such as growth factors, chemokines, cytokines, which exert anti-inflammatory, protective, and regenerative functions (for review see^8^). They have been reported to be effective in preventing articular cartilage and bone degradation and improving symptoms in pathologies such as osteoarthritis and rheumatoid arthritis.^9,10^ In HA, only 2 experimental studies are available. In a first approach, a gene and cell therapy-based treatment reported that intra-articular injection of factor VIII-overexpressing MSCs ameliorated HA in factor VIII-deficient mice.^11^ The second approach evaluated the intra-articular injection of MSCs in a rabbit model of induced HA.^12^ The results indicated no improvement of the mechanical properties of cartilage and no beneficial effect on inflammatory cytokines in the synovium, 38 days after MSC implantation. These disappointing results might be attributed to spontaneous cartilage repair that is known to occur in rabbits and to a lack of accurate techniques to evaluate cartilage regeneration and tissue homeostasis.

The objective of the present study was therefore to develop a relevant and reproducible in vitro model of hemarthrosis relying on blood exposure of primary murine chondrocytes to reproduce the main characteristics of blood-induced deleterious effects on cartilage cells. The final aim was to evaluate the potential therapeutic effects of MSCs in a model of blood-exposed chondrocytes mimicking hemarthrosis conditions close to the clinical situation.

## MATERIALS AND METHODS

### Chondrocyte culture and blood exposure

Murine articular chondrocytes were isolated from the knees and femoral heads of 5–7 days old Swiss mice as described.^13^ Chondrocytes were plated in TPP® tissue culture plates (TPP Techno Plastic Products, Switzerland) at 25,000 cells/cm^2^ in proliferative medium (Dulbecco's Modified Eagle Medium containing 10% fetal calf serum, 2 mmol/mL glutamine, and 100 µg/mL penicillin/streptomycin) for 3 days. Thereafter, proliferative medium with 10%, 20%, or 30% of fresh whole heparinized blood withdrawn from 3-week-old syngeneic mice and 50 µL/mL of sodium heparin at 5000 UI/mL for 2–4 days. The medium was resuspended daily to avoid coagulation at the bottom of the wells. After blood exposure, chondrocytes were rinsed 3 times with PBS to remove blood deposit and recovered for analysis.

### MSC culture

Murine MSCs were isolated from the bone marrow of C57BL/6J mice and characterized as previously described.^14^ A similar procedure was used to isolate MSCs from C57BL/6J mice knock-out for interleukin-6 (*Il6*), nitric oxide synthase 2 (*Nos2* or *iNos*), glucocorticoid-induced leucine zipper (*Gilz*), or from BALB/c mice knock-out for interleukin 1 receptor antagonist (*Il1ra*), thereafter called *Il6^−/−^, Il1ra^−/−^, Nos2^−/−^, Gilz^−/−^* MSCs, respectively.^14–16^ They were expanded in proliferative medium and used between passage 10 and 20. For blood exposure experiments, MSCs were plated at 3000 cells/cm^2^ in proliferative medium for 48 hours. The supernatant was replaced by the proliferative medium with 30% of heparinized whole blood freshly withdrawn from 3-week-old Swiss mice and 50 µL/mL of sodium heparin at 5000 UI/mL for 1, 2, or 4 days. MSCs were rinsed 3 times with PBS to remove blood deposit and recovered for analysis.

### Chondrocyte and MSC coculture

For coculture experiments, MSCs were seeded at 30,000 cells/cm^2^ in cell culture inserts (0.4-µm pore size, Transwell, Corning) in proliferative medium for 24 hours. MSCs-containing culture inserts were added onto chondrocytes plated in culture plates according to the different exposure conditions. In the preventive approach, MSCs-containing culture inserts were added to chondrocytes for 3 days and the inserts were removed before blood exposure of chondrocytes for an additional 4 days. In the concomitant approach, MSCs-containing inserts were added for 1, 2, or 4 days on top of the chondrocytes seeded in the bottom of the culture wells and the proliferative medium with 30% whole blood and 50 µL/mL of sodium heparin at 5000 UI/mL was added in the 2 compartments. In the therapeutic approach, MSCs-containing inserts were added to chondrocytes after blood exposure for 4 days and the coculture was maintained in proliferative medium for 1–3 additional days. After coculture, chondrocytes were rinsed 2 times with PBS and recovered for analysis.

### Survival and apoptosis assays

Cell survival was evaluated using the CellTiter-Glo Luminescent Cell Viability Assay (PROMEGA) and apoptosis was quantified using the Caspase-Glo 3/7 Assay (PROMEGA) according to the supplier’s recommendations. Survival rates are expressed as the percentage of viability related to the control (either non-exposed or blood-exposed chondrocytes) set at 100%. Apoptosis rates are expressed as the ratio of the luminescent signal obtained with the Caspase-Glo 3/7 assay normalized on the luminescent signal obtained with the CellTiter-Glo assay.

### Splenocyte proliferative assay

Splenocytes were isolated from C57BL/6J mice and cultured with MSCs as described.^17^ In brief, MSCs were exposed to the proliferative medium containing 30% of whole blood for 24 hours. Splenocytes were then added at 2 × 10^5^ cells/100 µL/well and mitogen-driven proliferation of T lymphocytes was induced by adding 5 µg/mL concanavalin A (Sigma-Aldrich, Saint-Quentin-Fallavier). After 3 days of incubation, splenocyte proliferation was assessed using the CellTiter-Glo Luminescent Cell Viability Assay (PROMEGA) according to the supplier’s recommendations. T cell proliferation was quantified by subtracting the signal of unstimulated splenocytes and expressed as the percentage of the value obtained with concanavalin A-stimulated splenocytes.

### RNA extraction and RT-qPCR

Total RNA was isolated from chondrocytes or MSCs using the RNeasy kit according to the supplier’s recommendations (Qiagen, Les Ulis). Total RNA (500 ng) was reverse transcribed by M-MLV reverse transcriptase (Thermo Fisher Scientific, Illkirch-Graffenstaden). Real-time PCR was done on 10 ng cDNA using the SYBR Green I Master mix (Roche Diagnostics, Meylan) and specific primers (Table 1). Values were normalized to the Ribosomal Protein S9 (RPS9) housekeeping gene and expressed as relative expression or fold change using the respective formulae: 2^− Δct^ or 2^− ΔΔct^.

**Table 1:** List of Primers for RT-qPCR

Gene	Forward Primer	Reverse Primer
*Rps9*	GCT GTT GAC GCT AGA CGA GA	ATC TTC AGG CCC AGG ATG TA
*Col2A1ΔB*	CTG GTG CTG CTG ACG CT	GCC CTA ATT TTC GGG CAT
*Agg*	GCG AGT CCA ACT CTT CAA GC	GAA GTA GCA GGG GAT GGT GA
*Mmp13*	TCT GGA TCA CTC CAA GGA CC	ATC AGG AAG CAT GAA ATG GC
*Adamts5*	CTG CCT TCA AGG CAA ATG TGT GG	CAA TGG CGG TAG GCA AAC TGC
*Tnfα*	AGC CCA CGT CGT AGC AAA CCA	TGT CTT TGA GAT CCA TGC CGT TGG C
*IL6*	TGG GAC TGA TGC TGG TGA CA	TTC CAC GAT TTC CCA GAG AAC A
*Nos2*	CCT TGT TCA GCT ACG CCT TC	GCT TGT CAC CAC CAG CAG TA
*Cox2*	CCA GCA CTT CAC CCA TCA GTT	ACC CAG GTC CTC GCT TAT GA
*Tgfβ*	TGC GCT TGC AGA GAT TAA AA	CTG CCG TAC AAC TCC AGT GA
*Il1-ra*	AGG CCC CAC CAC CAG CTT TGA	GGG GCT CTT CCG GTG TGT TGG T
*Hmox1*	GCA GAG CCG TCT CGA GCA TA	GCA TTC TCG GCT TGG ATG TG
*Sod2*	TCA GGA CCC ATT GCA AGG AA	TGT GGC CGT GAG TGA CGT TT
*Tsg6*	AAG CTC ACC TAC GCC GAA GC	TCC ATC CAG CAG CAC AGA CA

### Statistical analysis

Statistical analysis was conducted with the GraphPad Prism Software version 9.3.1. Data distribution and variance homogeneity were determined with the Shapiro-Wilk normality test. Comparison between groups was performed using the Wilcoxon signed-rank test when data were normalized on control and the Wilcoxon matched test or paired *t* test depending on data distribution otherwise. Data are presented as the mean ± SEM with *P* < 0.05 (* or #).

## RESULTS

### Hemarthrosis-related phenotypic changes in chondrocytes

**Figure 1.**
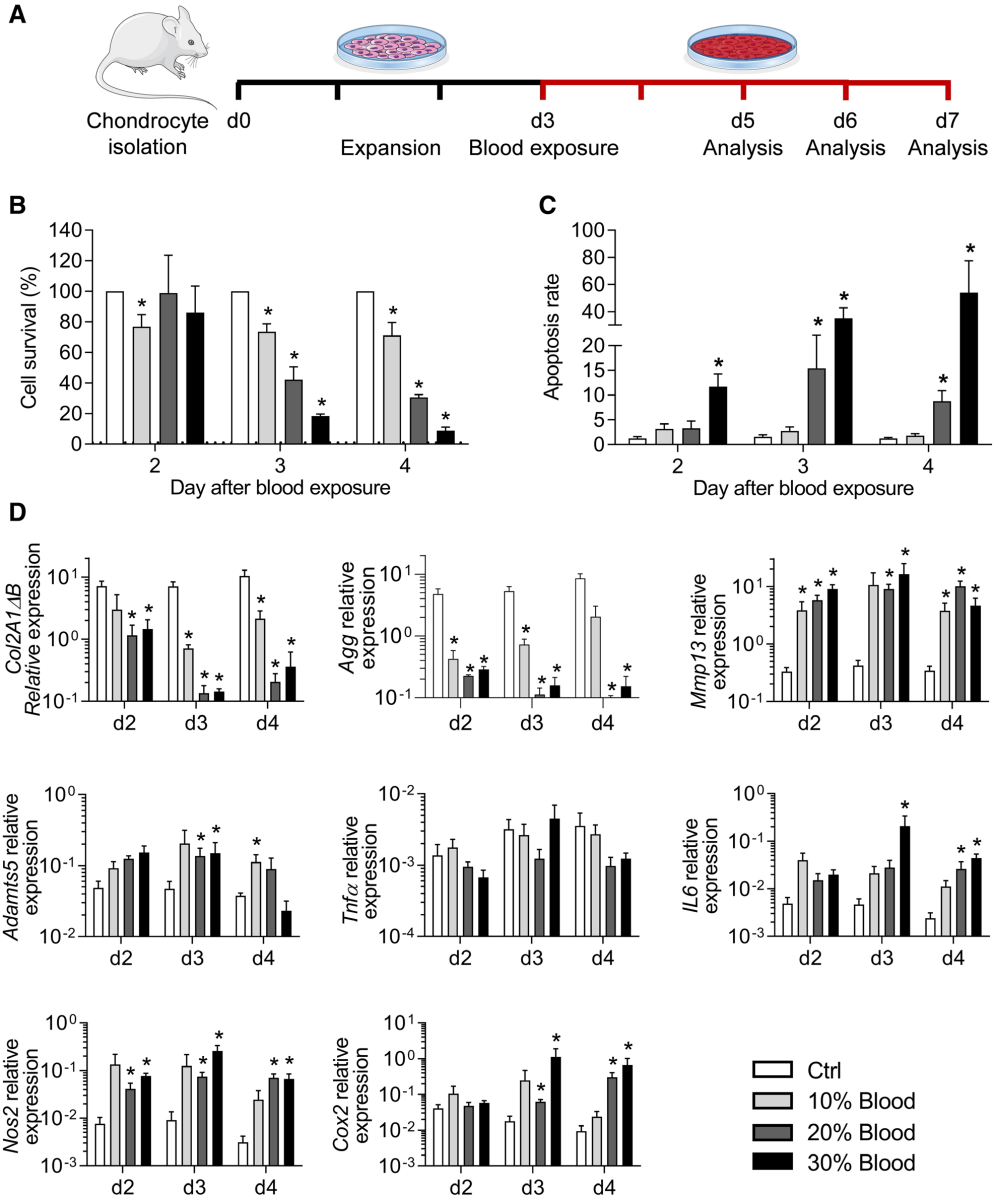
**Blood exposure of murine chondrocytes induced hemarthrosis-like degenerative features.** (A) Experimental scheme. Murine chondrocytes were isolated from articular cartilage of 5–7 days old mice and cultured for 3 days before exposure to different concentrations of blood (10%, 20%, 30%) and analysis. (B) Quantification of chondrocyte survival at different time points and expressed as the percentage of non-exposed control. (C) Quantification of the chondrocyte apoptosis rate at different time points (normalized on the number of alived cells). (D) Relative expression levels of genes related to anabolism, catabolism, inflammation, and oxidative stress in chondrocytes at different time points. Results are expressed as mean ± SEM (n = 4) with *: *P* < 0.05 vs nonexposed control group.

Our primary objective was to generate an in vitro model of hemarthrosis by culturing murine primary chondrocytes with whole blood. After chondrocyte isolation and expansion for 3 days, we evaluated the impact of different blood concentrations and exposure durations (2–4 days) on the survival and phenotype of chondrocytes (Figure 1A). The survival rate was not affected by the exposure to blood for the first 2 days (Figure 1B). By contrast, after 3 and 4 days of exposure, a dramatic decrease in the survival rate of chondrocytes was observed in a dose-dependent manner when cultured in medium containing 10%, 20%, or 30% whole blood. In parallel to the decrease in the survival rate, the apoptosis rate increased (Figure 1C). The apoptosis rate of chondrocytes was not modulated by the addition of 10% blood while it raised to around 10%–15% of apoptotic chondrocytes when cultured with 20% whole blood for 3 or 4 days. The addition of 30% blood significantly increased the apoptosis rate up to 15% at day 2 and reached 40%–50% when cultured for 3 or 4 days.

We then evaluated the effect of blood exposure on the chondrocyte phenotype. Expression of the anabolic markers, type IIB collagen (*Col2A1ΔB*) and aggrecan (*Agg*), was hugely and significantly decreased as soon as day 2 after exposure (Figure 1D). The highest decrease in expression was observed when chondrocytes were exposed to 20% or 30% blood by day 3 and 4. In parallel, the expression of catabolic markers increased dramatically for matrix metalloproteinase 13 (*Mmp13*) and to a lesser extent for a disintegrin and metalloproteinase with thrombospondin motifs 5 (*Adamts5*), whatever were the concentration or time of blood exposure. The expression of inflammatory mediators nitric oxide synthase 2 (*Nos2),* cyclooxygenase 2 *(Cox2*), and *Il6* was significantly increased after exposure to 20% or 30% of whole blood by day 3 and 4 while no effect was noticed on the expression of tumor necrosis factor α (*Tnfα*). In summary, a concentration of 30% whole blood for 4 days allowed to induce the characteristic features of hemarthrosis including low survival of chondrocytes, apoptosis induction, and dysregulation of chondrocyte markers in favor of a catabolic and inflammatory phenotype. These culture conditions were used in further experiments.

### MSCs protect chondrocytes from blood-induced damages

**Figure 2.**
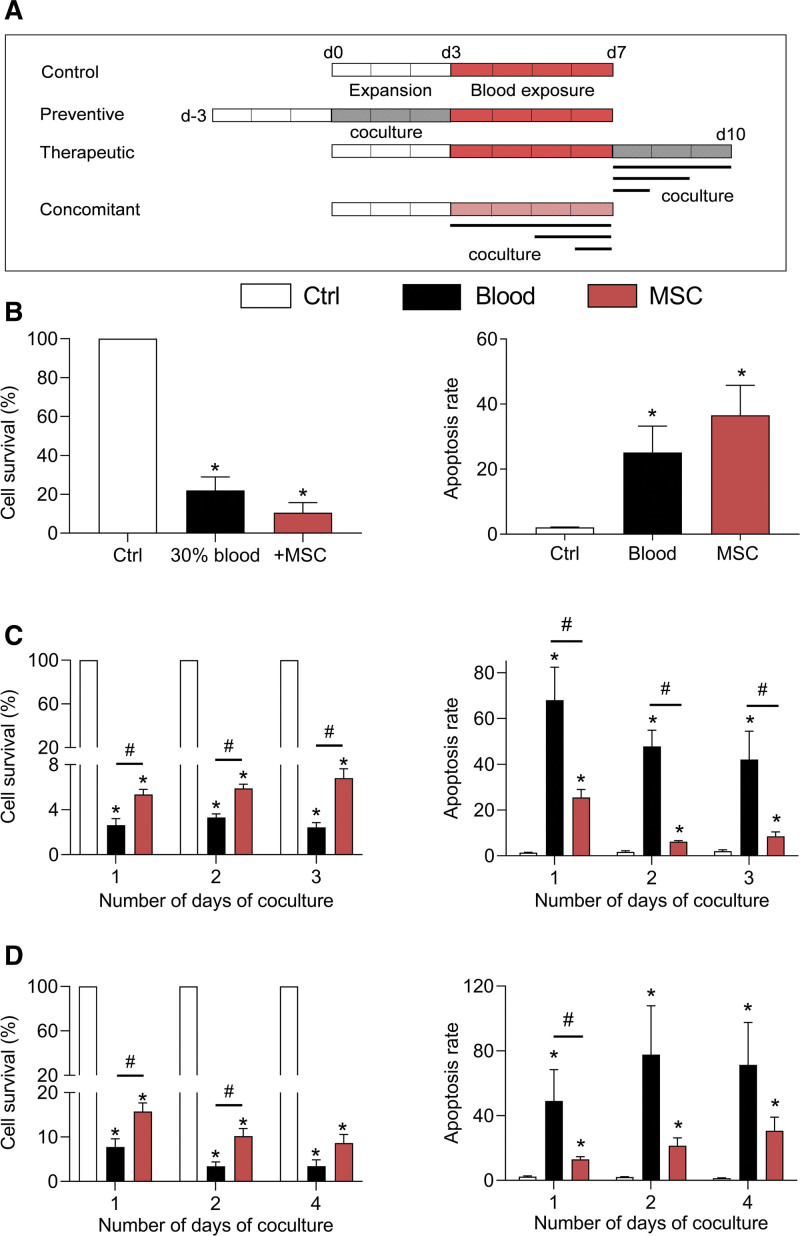
**MSCs improved the survival of murine chondrocytes exposed to blood.** (A) Experimental scheme. MSCs seeded on a culture insert were added on top of chondrocytes before (preventive approach), after (therapeutic approach), or during (concomitant approach) blood exposure, for 1–4 days. (B) Quantification of chondrocyte survival and apoptosis rates in the preventive approach, at day 10 (n = 5). (C) Quantification of chondrocyte survival and apoptosis rates in the therapeutic approach at day 10, after different exposure times with MSCs (n = 5). (D) Quantification of chondrocyte survival and apoptosis rates in the concomitant approach at day 7, after different exposure times with MSCs (n = 7). Results are expressed as mean ± SEM with *: *P* < 0.05 vs non-exposed control group. MSCs = mesenchymal stromal cells.

Using this in vitro model of hemarthrosis, we investigated whether MSCs can exert a beneficial effect on chondrocytes in terms of survival and phenotype. First, we tested a preventive approach where MSCs were cocultured with chondrocytes for 3 days and removed before blood addition on chondrocytes for 4 additional days (Figure 2A). At day 10, survival and apoptosis of chondrocytes were quantified. In these conditions, addition of MSCs before hemarthrosis induction did not improve the survival or apoptosis rate and even significantly reduced chondrocyte survival (Figure 2B).

We then investigated a therapeutic approach. Chondrocytes were cultured with blood for 4 days before the addition of MSCs and culture in a proliferative medium for 1–3 additional days, thereby mimicking bleeding resolution (Figure 2A). At day 10, chondrocytes that have been cultured with blood for 4 days experienced a dramatic decrease of survival while the apoptosis rate highly increased (Figure 2C). The addition of MSCs for 1–3 days after blood exposure partially but significantly prevented chondrocyte death as shown by higher survival percentage and lower apoptotic rate independently of the duration of coculture.

The next experiment investigated the effect of MSCs during the acute phase of hemarthrosis simulated by the concomitant exposure of chondrocytes and MSCs to blood. MSCs were cocultured with chondrocytes for 1–4 days during hemarthrosis induction (Figure 2A). At day 7 compared with hemarthrosis control, the percentage of chondrocytes that survived was higher when MSCs were present, whatever the number of coculture days (Figure 2D). These data mirrored the apoptosis rate, which was significantly decreased when chondrocytes were cocultured with MSCs. These results indicate that MSCs improved the survival of chondrocytes to the same extent when added either during the resolution or the acute phase.

**Figure 3.**
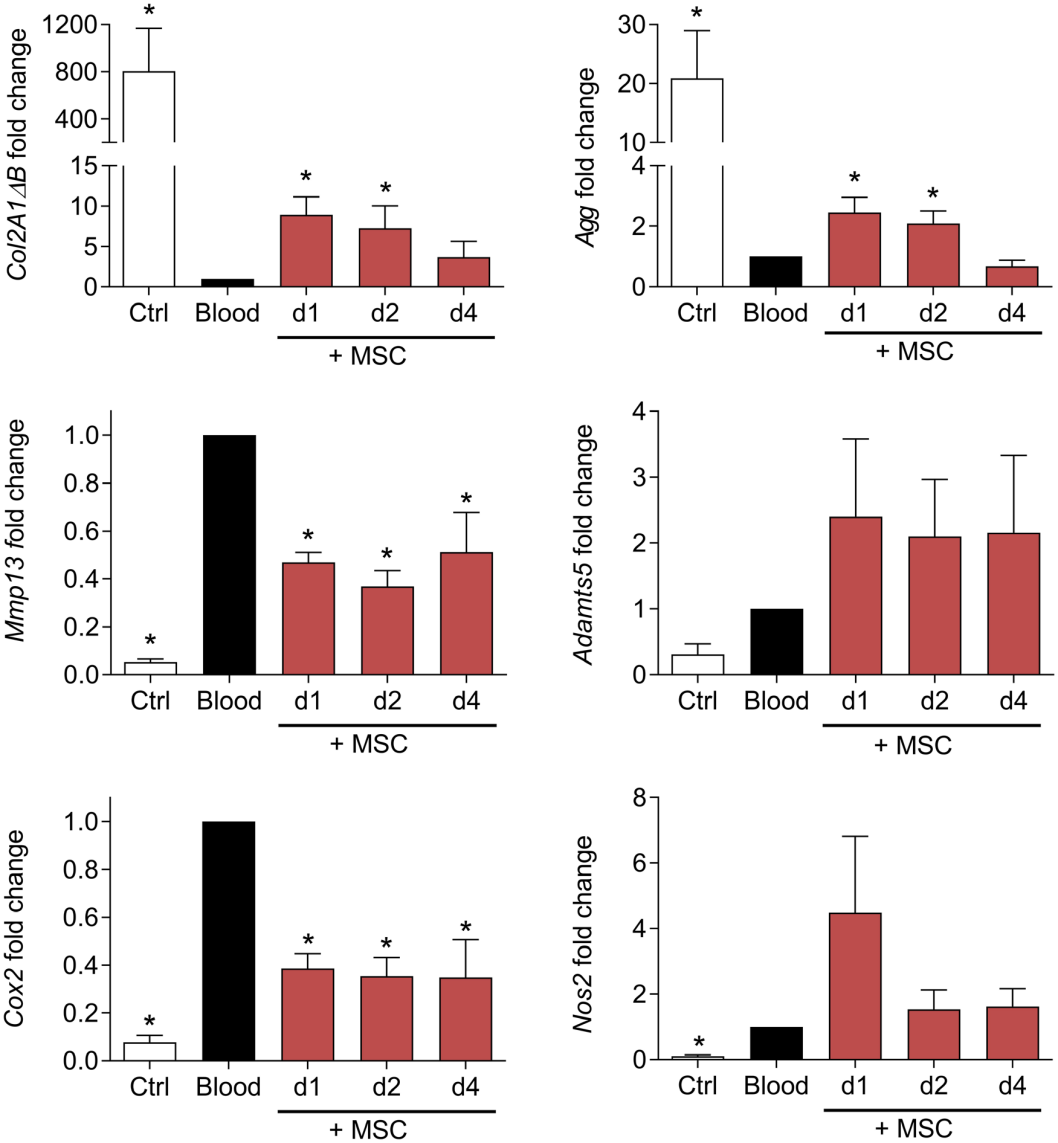
**MSCs improved the phenotype of chondrocytes exposed to blood.** Fold change expression levels of genes in chondrocytes at different time points of coculture with MSCs in the concomitant approach. Results are expressed as mean ± SEM (n = 6) with *: *P* < 0.05 vs non-exposed control group. MSCs = mesenchymal stromal cells.

Because we hypothesize that the therapeutic window of MSC-based treatment should focus on the acute phase of bleeding, which could last for several days, we investigated the impact of MSC coculture on chondrocyte phenotype in this condition (Figure 2D). We observed that MSCs maintained higher expression levels of anabolic markers (*Col2A1ΔB, Agg*) and repressed the expression of catabolic and inflammatory markers such as *Mmp13* and *Cox2* while the expression of *Adamts5* and *Nos2* was not significantly modulated (Figure 3). This chondroprotective effect of MSCs was seen, essentially on anabolic markers, when they were added in coculture for 1 or 2 days and disappeared for a 4 days coculture. This raised the question on the survival and function stability of MSCs that were exposed to blood.

### Blood exposure partially impairs MSC function

**Figure 4.**
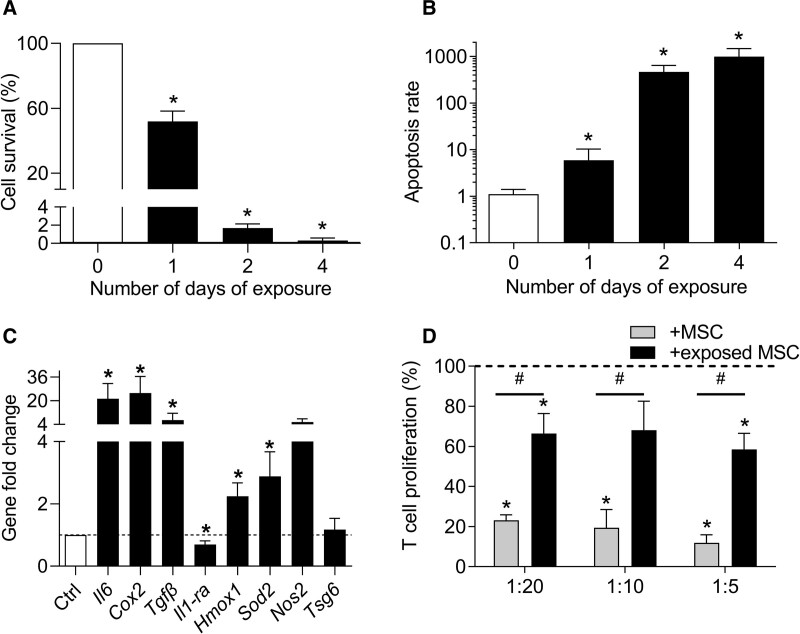
**Blood-exposed MSCs exhibited an impaired phenotype.** (A) Quantification of MSC survival at different time points after blood exposure expressed as the percentage of non-exposed control (n = 4). (B) Quantification of the MSC apoptosis rate at different time points (normalized on the number of alived cells) (n = 4). (C) Fold change expression levels of genes related to the immunosupressive function of MSCs at day 1 (n = 8). (D) Proliferation of T lymphocytes in presence of MSCs exposed or not to blood before addition in a mixed lymphocyte reaction (n = 6). Results are expressed as mean ± SEM with *: *P* < 0.05 vs non-exposed control group (A–C) or T lymphocytes cultured alone (D); #: *P* < 0.05 vs the indicated group. MSCs = mesenchymal stromal cells.

We therefore investigated the effect of total blood on the survival and function of MSCs. A time-dependent decrease of MSC survival was observed when they were exposed to 30% blood going from 50% at day 1 to 2% at day 2 and 0.3% at day 4 (Figure 4A). In parallel, the apoptosis rate significantly increased from day 1 to day 4, with a dramatic increase of apoptosis from day 2 (Figure 4B). After 24 hours of blood exposure, MSCs expressed higher levels of markers known to be involved in their anti-inflammatory function, including *Il6, Cox2, Tgfβ* as well as markers of oxidative stress regulation, such as *Hmox1, Sod2, Nos2* (Figure 4C). By contrast, the expression of *Il1-ra* was decreased while *Tsg6* expression was not modulated. These results indicated that the anti-inflammatory function of MSCs was highly upregulated by blood addition. We, therefore, tested the immunosuppressive activity of MSCs, which have been previously cultured in presence of 30% blood for 24 hours, in a proliferative assay. While naïve MSCs exerted a dose-dependent inhibitory effect on T lymphocyte proliferation, blood-exposed MSCs partially lost their immunosuppressive function compared with the proliferation rate of control T lymphocytes (Figure 4D). These data indicate that blood exposure impaired the survival and anti-inflammatory function of MSCs while preserving their regenerative secretory profile.

### The protective effect of mMSCs on murine chondrocytes is dependent of Nos2 and Il6

**Figure 5.**
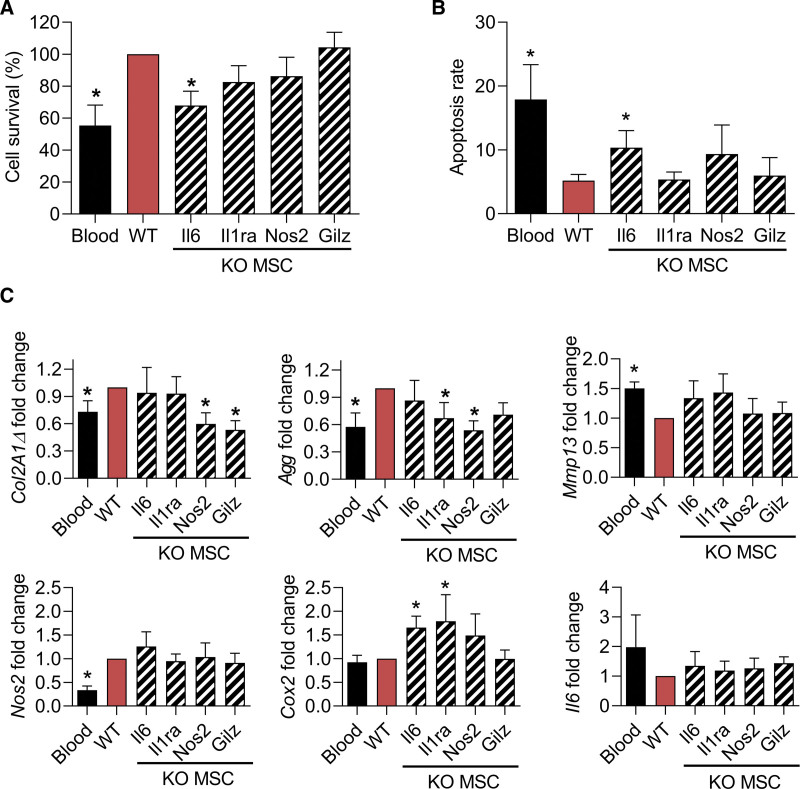
**Mechanism of action of MSCs for their chondroprotective effect.** (A) Quantification of chondrocyte survival at different time points after blood exposure and cultured in presence of WT MSCs or MSCs KO for the indicated immunosuppressive factors. Values are expressed as the percentage of survival vs the WT MSC group. (B) Quantification of the chondrocyte apoptosis rate at different time points after blood exposure and cultured in presence of WT or KO MSCs (normalized on the number of alived cells). (C) Fold change expression levels of genes related to anabolism, catabolism, inflammation, and oxidative stress in chondrocytes. Results are expressed as mean ± SEM (n=7) with *: *P* < 0.05 vs the WT MSC group. KO = knock-out; MSCs = mesenchymal stromal cells; WT = wild-type.

Finally, we evaluated whether one of the main factors that we have previously shown to play a role in the anti-inflammatory function of MSCs might be responsible of the protective effect of MSCs in this model of hemarthrosis. To this aim, we used MSCs deficient for *Il6, Il1-ra, Nos2*, or *Gilz* and cultured them with chondrocytes and 30% blood for 3 days.^14–16^ As previously observed, wild-type MSCs significantly enhanced the survival rate and decreased the apoptosis rate of chondrocytes (Figure 5A and 5B). By contrast, MSCs deficient for *Il6* lost this preservative function while the other deficient MSCs have no effect. When looking at the phenotype of chondrocytes, wild-type MSCs significantly upregulated the expression of *Col2A1ΔB*, *Agg*, *Nos2* and downregulated the expression of *Mmp13* but did not modulate the expression of *Cox2* and *Il6* (Figure 4C). *Nos2^-/-^* MSCs lost the capacity to upregulate the 2 anabolic markers while *IL1-ra*^*−/−*^ and *Gilz*^*−/−*^ MSCs did not upregulate the expression of *Agg* and *Col2A1ΔB,* respectively. Altogether, the data suggest that *Il6* secreted by MSCs is an important factor mediating the survival of chondrocytes while Nos2 might have a proanabolic effect of chondrocytes.

## DISCUSSION

In the present study, we generated a new model of hemarthrosis based on the culture of murine chondrocytes with whole blood, characterized by increased apoptosis and deleterious effects on the expression of chondrocyte markers, which are representative of the altered cartilage in patients with HA. Moreover, we demonstrated that a MSC-based therapy reduces apoptosis and, partially but significantly, prevents the downregulation of anabolic markers and the upregulation of catabolic and inflammatory markers.

To our knowledge, this is the first in vitro model of blood-induced hemarthrosis using isolated murine chondrocytes. Among the few studies that have used in vitro models of hemarthrosis, most of them used cartilage explants from healthy subjects^18–20^ or patients with osteoarthritis^21^ but none relied on isolated chondrocytes. Human cartilage explants were cultured with 10% (vol/vol) whole blood for 24 and 96 hours or, 50% (vol/vol) whole blood for 4 days. It is assumed that at least 50% blood is present in the joint shortly after bleeding and the time span before elimination is a 4-day period.^22^ In these models, the addition of blood induced lower proteoglycan synthesis and higher glycosaminoglycan release, indicating extracellular matrix degradation^18,20,23–25^ and upregulated the expression of IL1β, prostaglandin E2 (PGE2), and increased metalloproteinase (MMP) activity and the release of nitric oxide.^21^ In our study, we found a dose-dependent deleterious effect of whole blood on chondrocyte viability and phenotype alteration as soon as day 2. Indeed, blood exposure dramatically affected the expression of anabolic, catabolic, inflammatory, and oxidative stress markers in chondrocytes by day 3 and 4 upon addition of 20% or 30% of whole blood. A dose-dependent effect of blood on the inhibition of proteoglycan synthesis, which was only partially reversible, has already been reported.^19^ Here, we did not evaluate the impact of blood from hemophilic mice on chondrocyte viability and phenotype. Absence of factor VIII in blood might affect chondrocyte viability or phenotype because it has been reported to influence bone homeostasis, stimulating osteoclastogenesis and inducing apoptosis.^26^ As a matter of fact, we used heparinized blood to avoid coagulation and mimic the absence of factor VIII but the influence of heparin would deserve to be studied. In our conditions, addition of 30% of blood resulted in a huge decrease of viability (by 92%) by day 3. Preliminary experiments had indicated that 50% blood led to chondrocyte death within 2–3 days. Assessment of viability and apoptosis rates was not previously reported but we can correlate chondrocyte apoptosis with altered expression of chondrocyte markers. We therefore assume that viability and apoptosis rates could be additional relevant markers of hemarthrosis as proposed in one study.^20^ Our study validated the culture of murine primary chondrocytes with 30% whole blood for 4 days as the conditions of hemarthrosis induction in vitro.

We used this model to evaluate the therapeutic effect of murine MSCs on chondrocytes subjected to hemarthrosis using viability and apoptosis rates as preliminary read-outs of different approaches. Addition of MSCs in a preventive approach, before hemarthrosis induction, did not protect chondrocytes from apoptosis whereas addition of MSCs either in a therapeutic or concomitant approach exerted a protective effect on chondrocytes. Indeed, MSCs reduced by 2.5- to 8-fold the apoptosis rates depending on the duration of cocultures. Although not realistic from a clinical perspective, the preventive approach was evaluated to determine whether MSCs required activation by blood exposure to exert a therapeutic effect. And actually, the lack of beneficial effect is consistent with the fact that MSCs were not primed and could not modulate their secretome composition to counterbalance the effect of blood-induced deleterious environment.^27^ The concomitant approach could be considered as the most relevant in clinical practice where the treatment window should occur in the first 48 hours after bleeding to prevent or diminish long-lasting impairment of cartilage.^25^ In the concomitant approach, MSCs also improved the expression of chondrocyte anabolic markers when added for 1 or 2 days as well as catabolic and inflammatory markers, independently of the duration of coculture. Blood-induced priming of MSCs might explain the beneficial effect of MSCs on chondrocytes in these conditions. Similar therapeutic effect of MSCs has been reported for chondrocytes from patients with osteoarthritis or rheumatoid arthritis.^14,28–30^ However, the present results are the first proof-of-evidence that MSCs improve the phenotype of chondrocytes under hemarthrosis conditions. The only reports available to date on the effects of MSCs in hemarthrosis conditions indicated that MSCs genetically engineered to overexpress factor VIII ameliorated HA after intra-articular injection in FVIII-deficient mice.^11^ This improvement was related to the production of coagulation factor VIII in a cell-based and gene-based therapy strategy.

One possible limiting effect of the treatment was the blood-induced toxicity on MSCs, as shown by a dramatic reduction in MSC viability as soon as day 2 of exposure. However, 24 hours of blood exposure was sufficient to highly upregulate the expression of several markers known to be involved in their anti-inflammatory and antioxidative functions. These results suggest that short-term survival of MSCs is sufficient to initiate their therapeutic function and explain the beneficial effect observed on chondrocytes. Interestingly, we provide evidence that *IL-6* expression by MSCs is required to enhance chondrocyte survival. This is consistent with previous studies reporting that IL-6 produced by MSCs suppresses apoptosis but also upregulates PGE2 that induces anti-inflammatory M2 macrophages in a double therapeutic effect.^14,31^ Additionally, we showed that *Nos2* expression by MSCs increased the expression of anabolic markers *Col2A1Δ* and *Agg* whereas NO production is known to contribute to cartilage damage. We can speculate that the effect of *Nos2* is indirect by acting on other cellular processes that upregulate the anabolic activity of chondrocytes. Indeed, NO is an important regulator of heme oxygenase-1, which in turn reduces the expression of proinflammatory factors, metalloproteinases, and upregulates *Agg* and *Col2A1Δ* as described in canine chondrocytes.^32,33^ Finally, we showed that blood exposure reduced the immunosuppressive capacities of MSCs, which suggests that in vivo, this function might be impaired. Nevertheless, MSC administration has been reported to exert some beneficial effect on cartilage structure in an in vivo model of hemarthrosis.^12^

In summary, we demonstrated for the first time the benefit of using MSCs-based therapy to counteract the deleterial effect of blood on chondrocytes in a relevant in vitro model of hemarthrosis. The present study warrants confirmation in preclinical in vivo studies but might be a promising option for patients with hemophilic arthropathies.

## AUTHOR CONTRIBUTIONS

AT did experimental work, data analysis, and article writing. MM and CBG did experimental work and data analysis. NS, CB, and CJ did conception and design. DN did conception and design, data analysis, and article writing.

## DISCLOSURES

The authors have no conflict of interest to disclose.

## SOURCES OF FUNDING

We acknowledge funding support from the Inserm Institute, the University of Montpellier, the Association Française des Hémophiles, Coordination Médicale pour l’Etude et le Traitement des maladies Hémorragiques et constitutionnelles (CoMETH), CSL Behring, Swedish Orphan BIovitrum (SOBI). We also acknowledge the Agence Nationale pour la Recherche for support of the national infrastructure: “ECELLFRANCE: Development of a national adult mesenchymal stem cell based therapy platform” (PIA/ANR-11-INSB-005). The funding bodies played no role in the design of the study and collection, analysis, and interpretation of data and in writing the article.

## References

[1] Berntorp E Fischer K Hart DP. Haemophilia. Nat Rev Dis Primers. 2021;7:45. 34168126 10.1038/s41572-021-00278-x

[2] Gualtierotti R Solimeno LP Peyvandi F. Hemophilic arthropathy: current knowledge and future perspectives. J Thromb Haemost. 2021;19:2112–2121. 34197690 10.1111/jth.15444 PMC8456897

[3] Manco-Johnson M. Comparing prophylaxis with episodic treatment in haemophilia A: implications for clinical practice. Haemophilia. 2007;13(Suppl 2):4–9. 10.1111/j.1365-2516.2007.01499.x 17685917

[4] Osooli M Steen Carlsson K Baghaei F. The association between health utility and joint status among people with severe haemophilia A: findings from the KAPPA register. Haemophilia. 2017;23:e180–e187. 28393468 10.1111/hae.13231

[5] Buccheri E Avola M Vitale N. Haemophilic arthropathy: a narrative review on the use of intra-articular drugs for arthritis. Haemophilia. 2019;25:919–927. 31639263 10.1111/hae.13857

[6] Rodriguez-Merchan EC. Surgical approaches to hemophilic arthropathy. Blood Coagul Fibrinolysis. 2019;30(1S Suppl 1):S11–S13. 31517710 10.1097/MBC.0000000000000824

[7] Pulles AE Mastbergen SC Schutgens RE. Pathophysiology of hemophilic arthropathy and potential targets for therapy. Pharmacol Res. 2017;115:192–199. 27890816 10.1016/j.phrs.2016.11.032

[8] Maumus M Jorgensen C Noel D. Mesenchymal stem cells in regenerative medicine applied to rheumatic diseases: role of secretome and exosomes. Biochimie. 2013;95:2229–2234. 23685070 10.1016/j.biochi.2013.04.017

[9] Bouffi C Djouad F Mathieu M. Multipotent mesenchymal stromal cells and rheumatoid arthritis: risk or benefit? Rheumatology (Oxford). 2009;48:1185–1189. 19561159 10.1093/rheumatology/kep162

[10] Ruiz M Cosenza S Maumus M. Therapeutic application of mesenchymal stem cells in osteoarthritis. Expert Opin Biol Ther. 2015;16:33–42. 26413975 10.1517/14712598.2016.1093108

[11] Kashiwakura Y Ohmori T Mimuro J. Intra-articular injection of mesenchymal stem cells expressing coagulation factor ameliorates hemophilic arthropathy in factor VIII-deficient mice. J Thromb Haemost. 2012;10:1802–1813. 22784361 10.1111/j.1538-7836.2012.04851.x

[12] Ravanbod R Torkaman G Mophid M. Experimental study on the role of intra-articular injection of MSCs on cartilage regeneration in haemophilia. Haemophilia. 2015;21:693–701. 25916559 10.1111/hae.12659

[13] Gosset M Berenbaum F Thirion S. Primary culture and phenotyping of murine chondrocytes. Nat Protoc. 2008;3:1253–1260. 18714293 10.1038/nprot.2008.95

[14] Bouffi C Bony C Courties G. IL-6-dependent PGE2 secretion by mesenchymal stem cells inhibits local inflammation in experimental arthritis. PLoS One. 2010;5:e14247. 21151872 10.1371/journal.pone.0014247 PMC2998425

[15] Luz-Crawford P Djouad F Toupet K. Mesenchymal stem cell-derived interleukin 1 receptor antagonist promotes macrophage polarization and inhibits B cell differentiation. Stem Cells. 2016;34:483–492. 26661518 10.1002/stem.2254

[16] Luz-Crawford P Tejedor G Mausset-Bonnefont AL. Gilz governs the therapeutic potential of mesenchymal stem cells by inducing a switch from pathogenic to regulatory Th17 cells. Arthritis Rheumatol. 2015;67:1514–1524. 25708718 10.1002/art.39069

[17] Maumus M Manferdini C Toupet K. Thrombospondin-1 partly mediates the cartilage protective effect of adipose-derived mesenchymal stem cells in osteoarthritis. Front Immunol. 2017;8:1638. 29238343 10.3389/fimmu.2017.01638 PMC5712679

[18] van Vulpen LFD Popov-Celeketic J van Meegeren MER. A fusion protein of interleukin-4 and interleukin-10 protects against blood-induced cartilage damage in vitro and in vivo. J Thromb Haemost. 2017;15:1788–1798. 28696534 10.1111/jth.13778

[19] Roosendaal G Vianen ME Marx JJ. Blood-induced joint damage: a human in vitro study. Arthritis Rheum. 1999;42:1025–1032. 10323460 10.1002/1529-0131(199905)42:5<1025::AID-ANR23>3.0.CO;2-3

[20] Hooiveld M Roosendaal G Wenting M. Short-term exposure of cartilage to blood results in chondrocyte apoptosis. Am J Pathol. 2003;162:943–951. 12598327 10.1016/S0002-9440(10)63889-8 PMC1868108

[21] Joos H Leucht F Riegger J. Brenner, differential interactive effects of cartilage traumatization and blood exposure in vitro and in vivo. Am J Sports Med. 2015;43:2822–2832. 26362437 10.1177/0363546515602248

[22] Jansen NW Roosendaal G Hooiveld MJ. Interleukin-10 protects against blood-induced joint damage. Br J Haematol. 2008;142:953–961. 18637801 10.1111/j.1365-2141.2008.07278.x

[23] Hooiveld M Roosendaal G Vianen M. Blood-induced joint damage: longterm effects in vitro and in vivo. J Rheumatol. 2003;30:339–344. 12563692

[24] Hooiveld MJ Roosendaal G Vianen ME. Immature articular cartilage is more susceptible to blood-induced damage than mature articular cartilage: an in vivo animal study. Arthritis Rheum. 2003;48:396–403. 12571849 10.1002/art.10769

[25] Jansen NW Roosendaal G Bijlsma JW. Exposure of human cartilage tissue to low concentrations of blood for a short period of time leads to prolonged cartilage damage: an in vitro study. Arthritis Rheum. 2007;56:199–207. 17195222 10.1002/art.22304

[26] Baud’huin M Duplomb L Teletchea S. Factor VIII-von Willebrand factor complex inhibits osteoclastogenesis and controls cell survival. J Biol Chem. 2009;284:31704–31713. 19758994 10.1074/jbc.M109.030312 PMC2797241

[27] Ferreira JR Teixeira GQ Santos SG. Mesenchymal stromal cell secretome: influencing therapeutic potential by cellular pre-conditioning. Front Immunol. 2018;9:2837. 30564236 10.3389/fimmu.2018.02837 PMC6288292

[28] Manferdini C Maumus M Gabusi E. Adipose-derived mesenchymal stem cells exert antiinflammatory effects on chondrocytes and synoviocytes from osteoarthritis patients through prostaglandin e2. Arthritis Rheum. 2013;65:1271–1281. 23613363 10.1002/art.37908

[29] Maumus M Manferdini C Toupet K. Adipose mesenchymal stem cells protect chondrocytes from degeneration associated with osteoarthritis. Stem Cell Res. 2013;11:834–844. 23811540 10.1016/j.scr.2013.05.008

[30] Ruiz M Toupet K Maumus M. TGFBI secreted by mesenchymal stromal cells ameliorates osteoarthritis and is detected in extracellular vesicles. Biomaterials. 2020;226:119544. 31648137 10.1016/j.biomaterials.2019.119544

[31] Gu Y He M Zhou X. Endogenous IL-6 of mesenchymal stem cell improves behavioral outcome of hypoxic-ischemic brain damage neonatal rats by supressing apoptosis in astrocyte. Sci Rep. 2016;6:18587. 26766745 10.1038/srep18587 PMC4725911

[32] Koike A Minamiguchi I Fujimori K. Nitric oxide is an important regulator of heme oxygenase-1 expression in the lipopolysaccharide and interferon-gamma-treated murine macrophage-like cell line J774.1/JA-4. Biol Pharm Bull. 2015;38:7–16. 25744452 10.1248/bpb.b14-00405

[33] Oh J Son YS Kim WH. Mesenchymal stem cells genetically engineered to express platelet-derived growth factor and heme oxygenase-1 ameliorate osteoarthritis in a canine model. J Orthop Surg Res. 2021;16:43. 33430899 10.1186/s13018-020-02178-4 PMC7802278

